# Alterations and Potential Applications of Gut Microbiota in Biological Therapy for Inflammatory Bowel Diseases

**DOI:** 10.3389/fphar.2022.906419

**Published:** 2022-06-06

**Authors:** Dan Pu, Zhe Zhang, Baisui Feng

**Affiliations:** Department of Gastroenterology, The Second Affiliated Hospital of Zhengzhou University, Zhengzhou, China

**Keywords:** gut microbiota, inflammatory bowel disease, biologic agents, faecal microbiota transplantation, biomarkers, combination therapy

## Abstract

Inflammatory bowel disease (IBD), including Crohn’s disease and ulcerative colitis, is a chronic immune-mediated inflammatory disorder of the gastrointestinal tract that is closely associated with dysbiosis of the intestinal microbiota. Currently, biologic agents are the mainstream therapies for IBD. With the increasing incidence of IBD, limitations of biologic agents have gradually emerged during treatment. Recent studies have indicated that gut microbiota is highly correlated with the efficacy of biologic agents. This review focuses on alterations in both the components and metabolites of gut microbiota during biological therapy for IBD, systematically summarises the specific gut microbiota closely related to the clinical efficacy, and compares current predictive models for the efficacy of biologics, further highlighting the predictive value of intestinal microbiota. Based on the mechanistic analysis of faecal microbiota transplantation (FMT) and biologic agents, a new therapeutic strategy, comprising a combination of FMT and biologics, has been proposed as a promising treatment for IBD with improved efficacy.

## 1 Introduction

Inflammatory bowel disease (IBD) refers to a group of immune-mediated inflammatory diseases of the intestinal tract that are associated with a variety of factors, including risk variants in the human genome, exposure to adverse environmental factors, and dysbiosis of the intestinal microbiome (Chu et al., 2016; Ni et al., 2017). IBD can be clinically phenotyped as Crohn’s disease (CD) and ulcerative colitis (UC) (Yilmaz et al., 2019). The second half of the 20th century witnessed a rapid increase in IBD morbidity in North America and Europe, along with a significant increase in Asian countries (Ananthakrishnan, 2015). Currently, IBD is a social burden of global concern (Chi, 2016).

Recently, biologic agents targeting tumour necrosis factor-alpha (TNFα), adhesion molecules, and the p40 subunit of interleukin (IL)-12/23 have emerged to revolutionise the treatment of IBD (Yanai and Hanauer, 2011; Feagan et al., 2013; Sands et al., 2019a). However, approximately 30% of patients with IBD still exhibit primary non-response during biological therapy (Stidham et al., 2014; Li et al., 2022), and secondary loss of response occurs in another 40% of patients with IBD (Kopylov et al., 2019; Argollo et al., 2020; Caenepeel et al., 2020). The main causes of failure to respond are the formation of antidrug antibodies (Bar-Yoseph et al., 2019; Sazonovs et al., 2020; Bots et al., 2021), drug immunogenicity (Yanai et al., 2015; Sazonovs et al., 2020; Srinivasan et al., 2021), and altered pharmacokinetics (Brandse et al., 2017). Additionally, side effects of biologic therapy, including the development of various infections, increased risk of tumours, and worsening of other autoimmune diseases, skin diseases, cardiac and neurological disorders, raise much distress, especially in patients who are also administered immunomodulators (Baddley et al., 2014; Shivaji et al., 2019).

Simultaneously, the therapeutic goals of IBD have gradually increased from clinical steroid-free remission to endoscopic remission and mucosal healing (Caenepeel et al., 2020; Kobayashi et al., 2020). Therefore, scientific and rational stratification of patients with IBD and the quest for personalised biological therapy strategies to maximise efficacy and minimise drug side effects are urgently needed in the field of IBD (Flamant and Roblin, 2018).

## 2 High Correlation Between Dysbiosis of Gut Microbiota and IBD

It is well-accepted that IBD results from altered interactions between gut microbiota and the mucosal immune system (Kostic et al., 2014). The anomalous immune response of the intestine, as the characteristic of IBD, correlates with dysbiosis of gut microbiota. At the genetic level, variants of NOD2 and ATG16L1, the main risk genes for IBD, cause defects in the innate immunity of the intestine, resulting in alterations in the structure of intestinal microbes and impaired protective function of commensal bacteria, thereby inducing the development of IBD (Chu et al., 2016). Numerous animal studies have confirmed that commensal bacteria maintain mucosal homeostasis by suppressing pathogenic innate and adaptive immune responses, inducing the secretion of antimicrobial peptides, and promoting epithelial restitution. Dysbiosis of the gut microbiota impairs the protective effects of healthy intestinal microecology, leading to increased immune stimulation, epithelial dysfunction, and enhanced mucosal permeability, all of which are associated with the development and dissemination of IBD (Sartor, 2008). Additionally, in real-world studies, alterations in the composition and metabolism of gut microbiota are being revealed in patients with IBD, further confirming the strong correlation between dysbiosis of gut microbiota and IBD.

### 2.1 Changes in the Composition of Gut Microbiota in Patients With IBD

Continuous updating of gene-sequencing technologies and the increased availability of powerful bioinformatics tools have provided a wealth of novel insights into the effect of microbial communities on IBD. Using these technologies, researchers have indicated that dysbiosis and decreased complexity of the gut microbial ecosystem are common features of patients with IBD (Manichanh et al., 2012). Changes in the gut microbiota of patients with IBD, namely, a significant decrease in commensal bacteria, were first reported in 2007 (Frank et al., 2007). Compared to healthy individuals, the gut microbiota’s α-diversity in patients with IBD is reduced by 50–70%, suggesting a serious imbalance in intestinal microecology (Ott et al., 2004). This imbalance includes a noticeable decrease in anti-inflammatory bacteria and a marked increase in pro-inflammatory opportunistic pathogens (Kang et al., 2010; Kostic et al., 2014; Kolho et al., 2015; Ribaldone et al., 2019).

Dysbiosis is more severe in patients with IBD during the active stage (Kolho et al., 2015), characterized by the increased abundance of Actinobacteria and Proteobacteria and decreased abundance of Firmicutes, all of which are strongly correlated with disease severity (Zhou et al., 2018). In addition, patients with different types of disease severity also have different distribution features of gut microbiota (Shaw et al., 2016). A higher proportion of Bacilli enrichment, represented by *Streptococcus*, have been detected in patients with mild CD. However, patients with severe CD demonstrated a significant increase in Proteobacteria and Enterococcaceae, but a remarkable decrease in Ruminococcaceae and Clostridiales. Correspondingly, the gut microbiota of patients with moderate UC is similar to that of patients with mild CD, showing an increased abundance of *Streptococcus*, whereas patients with severe UC have significant enrichment of Proteobacteria and Bacilli in their intestine (Zhou et al., 2018).

### 2.2 Alterations in Metabolism Associated With the Intestinal Microbiota in Patients With IBD

Along with compositional alterations, the metabolic function associated with the gut microbiota also changed notably, including decreased synthesis of short-chain fatty acids (SCFAs) and medium-chain fatty acids (MCFA), decreased amino acid biosynthesis, enrichment of acylcarnitines and disturbances of bile acid metabolism in IBD patients (De Preter et al., 2015; Jacobs et al., 2016; Franzosa et al., 2019; Lloyd-Price et al., 2019). In parallel, metabolic pathways, such as amino acid transport, sulfate transport, oxidative stress, and the type II secretory system (T2SS) are activated significantly in patients with IBD (Kostic et al., 2014). The current findings support a close correlation between these metabolic alterations and the pathological process of IBD.

SCFAs, which are products of bacterial fermentation in the gut, are of great value in modulating the host’s immune system. By targeting histone deacetylases and G protein-coupled receptors on intestinal epithelial cells or immune cells, SCFAs contribute to regulating cellular anti-inflammatory activity and regulatory T (Treg) cell development (Sun et al., 2017; Dupraz et al., 2021). Severe dysbiosis of the gut microbiota in patients with IBD results in reduced SCFA production, which consequently impairs protective intestinal immunity and exacerbates intestinal inflammation.

Recently, bile acids (BAs), as a key class of microbiota-associated metabolites in patients with IBD, have drawn considerable attention. In stool samples from patients with IBD, it was noted that primary BAs and conjugated BAs are increased while the secondary BAs reduced significantly. Previous researches have ascribed these changes to alterations in BAs absorptions, synthesis and bacterial modification (Li et al., 2021a). Furthermore, these characteristic alterations of BAs are considered to correlate with the disease severity of IBD (Duboc et al., 2013; Kriaa et al., 2022). Recent studies have also provided evidence for the involvement of BAs in IBD-associated autophagy, apoptosis, and inflammasome pathways (Thomas et al., 2022). Meanwhile, the interactions of BAs with intestinal epithelial cells and immune cells have also been revealed, further providing novel insights into the gut microbiota-BA-host axis in the pathogenesis of IBD (Yang et al., 2021; Kriaa et al., 2022).

Consequently, gut microbiota show marked compositional differences between populations with and those without IBD, between active and remissive phases, and between different disease severities, and exhibits significant alterations in intestinal metabolic activities. Current evidence has established that both composition and metabolism of gut microbiota are closely associated with the pathogenesis of IBD. Along with the emergence of functional metagenomic tools, the relationship between gut microbiota and IBD will be better elucidated. Moreover, high-throughput analysis of gut microbial composition and metabolism in large samples of patients with IBD to screen out specific gut microbiota and metabolic pathways will bring new biomarkers for the diagnosis of IBD and provide new perspectives for the exploration of therapeutic strategy (Dubinsky and Braun, 2015).

## 3 Alterations of Gut Microbiota Correlate With the Efficacy of Biological Therapy

**FIGURE 1:**
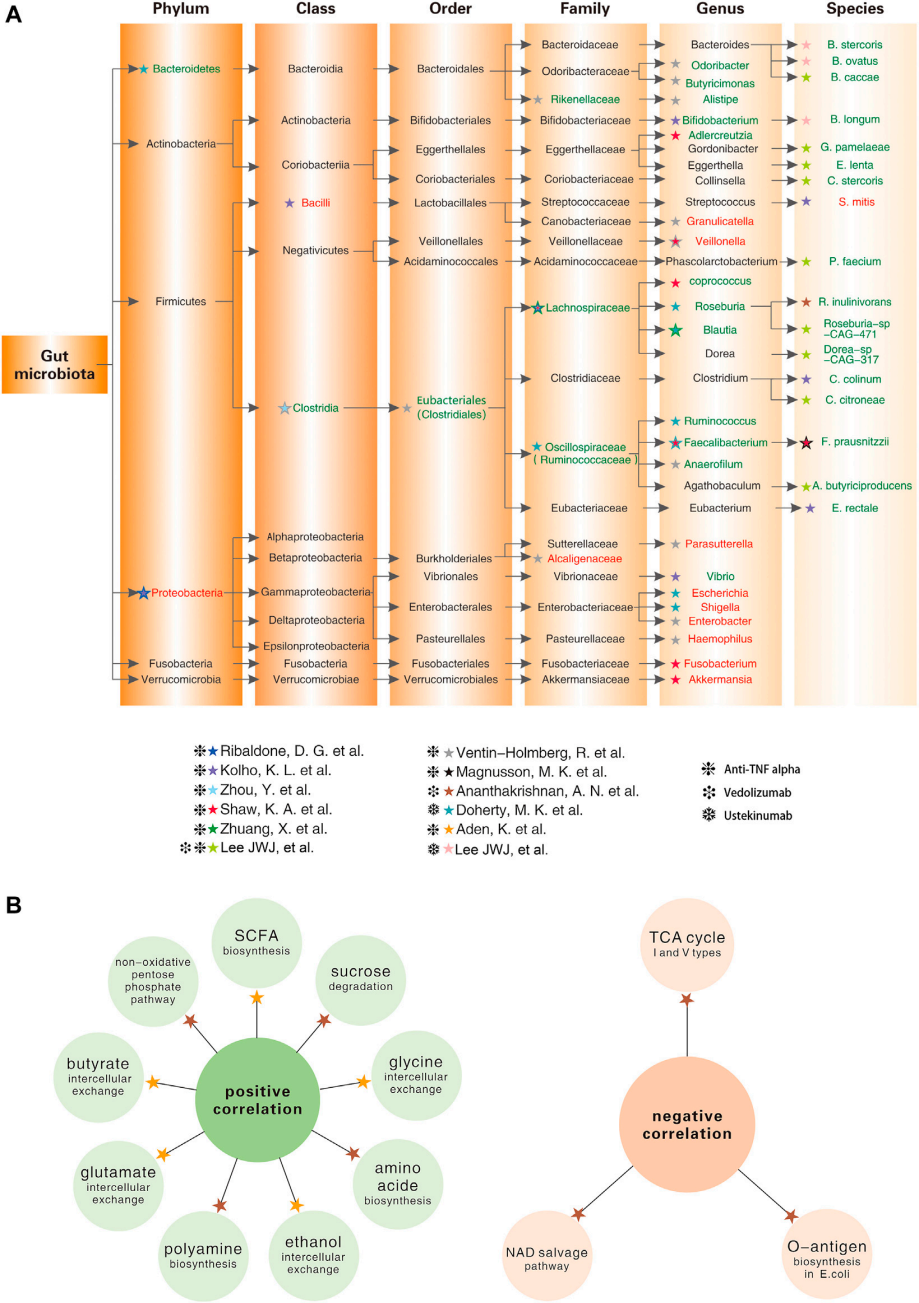
Baseline levels of gut microbial taxa and intestinal metabolism in IBD patients associate with different responses to biologic agents (Kolho et al., 2015; Magnusson et al., 2016; Shaw et al., 2016; Ananthakrishnan et al., 2017; Doherty et al., 2018; Zhou et al., 2018; Aden et al., 2019; Ribaldone et al., 2019; Zhuang et al., 2020; Lee et al., 2021; Ventin-Holmberg et al., 2021). **(A)** Lineage diagram showing the baseline abundance of specific gut microbes positively (in green font) or negatively (in red font) correlated with the good response to biologic agents; **(B)** The left panel shows intestinal metabolites that are positively correlated with the good response to the biologic agents; the right panel shows intestinal metabolites that are negatively correlated with the good response to the biologic agents.

In addition to infliximab, adalimumab, golimumab, and sacituzumab, all of which target tumour necrosis factor-alpha (TNFα), a growing number of biologics are used in the clinical treatment of IBD, such as ustekinumab that targets the shared p40 subunit of IL-12/23 and vedolizumab that target the alpha4beta7 integrin, etc. (Danese et al., 2015; Coskun et al., 2017; Moschen et al., 2019). Numerous studies have illustrated that the structure and function of gut microbiota changes significantly during biological therapy of IBD. Meanwhile, the compositional and functional metabolic levels of gut microbiome also differ at baseline corresponding to the different responses to biologic agents. (Figure 1).

### 3.1 Compositional Changes of Gut Microbiota During Biological Therapy

#### 3.1.1 Anti-TNFα Monoclonal Antibodies

Anti-TNFα monoclonal antibodies, especially infliximab, have been used as first-line therapy for IBD over the past 20 years. In recent years, the correlation between anti-TNFα therapy and gut microbiota has gradually become an important topic.

Anti-TNFα agents, such as infliximab and adalimumab, reduce disease severity and increase the alpha diversity of gut microbiota in patients with IBD over a short period (Estevinho et al., 2020). These agents result in a healthier gut microbiome composition, which is more pronounced in patients who respond well to biologics (Busquets et al., 2015; Zhou et al., 2018). A study that combined a Chinese IBD cohort and two western cohorts, PRISM and RISK, revealed that decreased levels of Clostridiales in the gut, as a hallmark of IBD, were significantly restored after infliximab therapy, and the baseline abundance of Clostridiales was positively associated with a good response to infliximab (Zhou et al., 2018). Recently, a similar study from Finland that included a larger sample size described in more detail the baseline groups that were positively or negatively associated with infliximab response at the family and genus levels (Ventin-Holmberg et al., 2021). In addition, separate studies have been conducted on different disease subtypes of IBD, such as CD and UC. A prospective study from Italy analysed changes in the intestinal microbiota during adalimumab treatment. It concluded that Proteobacteria decreased significantly and Lachnospiraceae increased in patients with CD who achieved remission after 6 months of adalimumab treatment, revealing a high correlation between the abundance of these two bacteria and therapeutic success (Ribaldone et al., 2019). In patients with UC, the abundance of *Faecalibacterium prausnitzii* in faecal samples have been demonstrated to be much higher in responders than non-responders at different time points during anti-TNFα therapy, even though the degree of gut microbiota dysbiosis varies considerably (Magnusson et al., 2016).

Similar to adults, altered gut microbiota diversity is also closely associated with the response to biologic therapies for paediatric inflammatory bowel disease as well (Kowalska-Duplaga et al., 2020). During the induction period of anti-TNFα therapy, the microbial diversity of the well-responding group increased in a similar manner as that of healthy individuals, whereas no such changes occurred in the non-responding group. At the species and genus levels, many sub-strains of Bacilli and Proteobacteria were enriched in the intestines of non-responders, as opposed to responders. Meanwhile, several groups of bacteria associated with anti-TNFα efficacy were identified, namely high abundance of Bifidobacterium, *Clostridium colinum, Eubacterium rectale*, and *Vibrio*, as well as low abundance of *Streptococcus mitis* at baseline were associated with good responses (Kolho et al., 2015).

In summary, anti-TNFα therapy reduces disease severity and increased gut microbial alpha diversity in patients with IBD (Zhou et al., 2018). More meaningfully, the abundance of specific gut microbes are strongly associated with the anti-TNFα response (Figure 1A).

#### 3.1.2 Other Biologic Agents

Vedolizumab, an intestine-selective humanised monoclonal antibody against alpha4beta7 integrin, has shown durable efficacy and high safety in the treatment of UC and CD (Feagan et al., 2013; Colombel et al., 2017; Sands et al., 2019b). In 2017, a study on the intestinal microbiota of CD patients who achieved 14-weeks remission with vedolizumab concluded that remitters possessed higher alpha-diversity of gut microbiota at baseline. Specifically, the abundance of *Roseburia inulinivorans* and Burkholderiales was markedly higher among patients with CD who achieved remission, whereas *Streptococcus salivarium* was enriched in patients with UC who did not achieve remission (Ananthakrishnan et al., 2017). More recently, a study incorporating a larger sample size revealed that three groups of bacteria, namely *Bifidobacterium longum* and two species of the genera Bacteroide, showed a marked positive correlation with remission at week 14 in patients with IBD treated with Vedolizumab (Lee et al., 2021). Similarly, a significant increase in microbiota diversity was also identified during the first 22 weeks of ustekinumab therapy, and differences in community diversity at baseline were strongly correlated with treatment response, especially in the genera *Faecalibacterium* and *Bacteroides* (Doherty et al., 2018) (Figure 1A).

### 3.2 Altered Intestinal Metabolism in Patients With IBD During Biological Therapy

Apart from compositional changes, there were also remarkable alterations in the metabolism of the gut microbiota during biological therapy. As discussed above, SCFA, a metabolite of the gut microbiota, regulates intestinal transit, nutrient absorption, intestinal pH, and immune function of colonic Treg cells, all of which are beneficial for reversing the inflammatory intestinal environment (Smith et al., 2013). Many SCFA-producers, including *Anaerostipes, Blautia, Coprococcus, Faecalibacterium, Lachnospira, Odoribacter, Roseburia, Ruminococcus* and *Sutterella*, are reduced in patients with IBD and are associated with disease relapse and poor anti-TNFα response (Wang et al., 2018; Yilmaz et al., 2019; Ventin-Holmberg et al., 2021).

In addition to the above changes in fatty acid metabolism, amino acid metabolism, folate biosynthesis, and signalling pathways are disrupted in patients with IBD (Kolho et al., 2017). In baseline samples from patients with CD who achieve remission after week 14 of anti-integrin therapy (vedolizumab), 13 metabolic pathways, including branched-chain amino acid synthesis, were significantly enriched, and reductions in several tricarboxylic acid cyclic (TC) pathways (I and V types) and the nicotinamide adenine dinucleotide (NAD) salvage pathway were also detected, suggesting reduced levels of oxidative stress (Ananthakrishnan et al., 2017). A prospective German study showed that metabolite exchange in faecal samples from patients with IBD was dramatically reduced at baseline and was associated with later clinical remission. At the same time, anti-TNFα treatment restored the disrupted gut microbial metabolism, including ethanol, glutamate, and glycine, none of which was observed in non-remitters (Aden et al., 2019) (Figure 1B).

In summary, both the composition and metabolism of the intestinal microbiota changed during biological therapy, and closely correlated with the response to biologics. This conclusion further supports the potential of the gut microbiota as a promising biomarker for predicting the efficacy of biological therapy for IBD.

## 4 Potential Application of Gut Microbiota in the Biological Therapy for IBD

### 4.1 Gut Microbiota as Biomarkers for the Efficacy of Biologic Agents

#### 4.1.1 Early Attempts to Seek Biomarkers for the Efficacy of Biologic Agents

**FIGURE 2:**
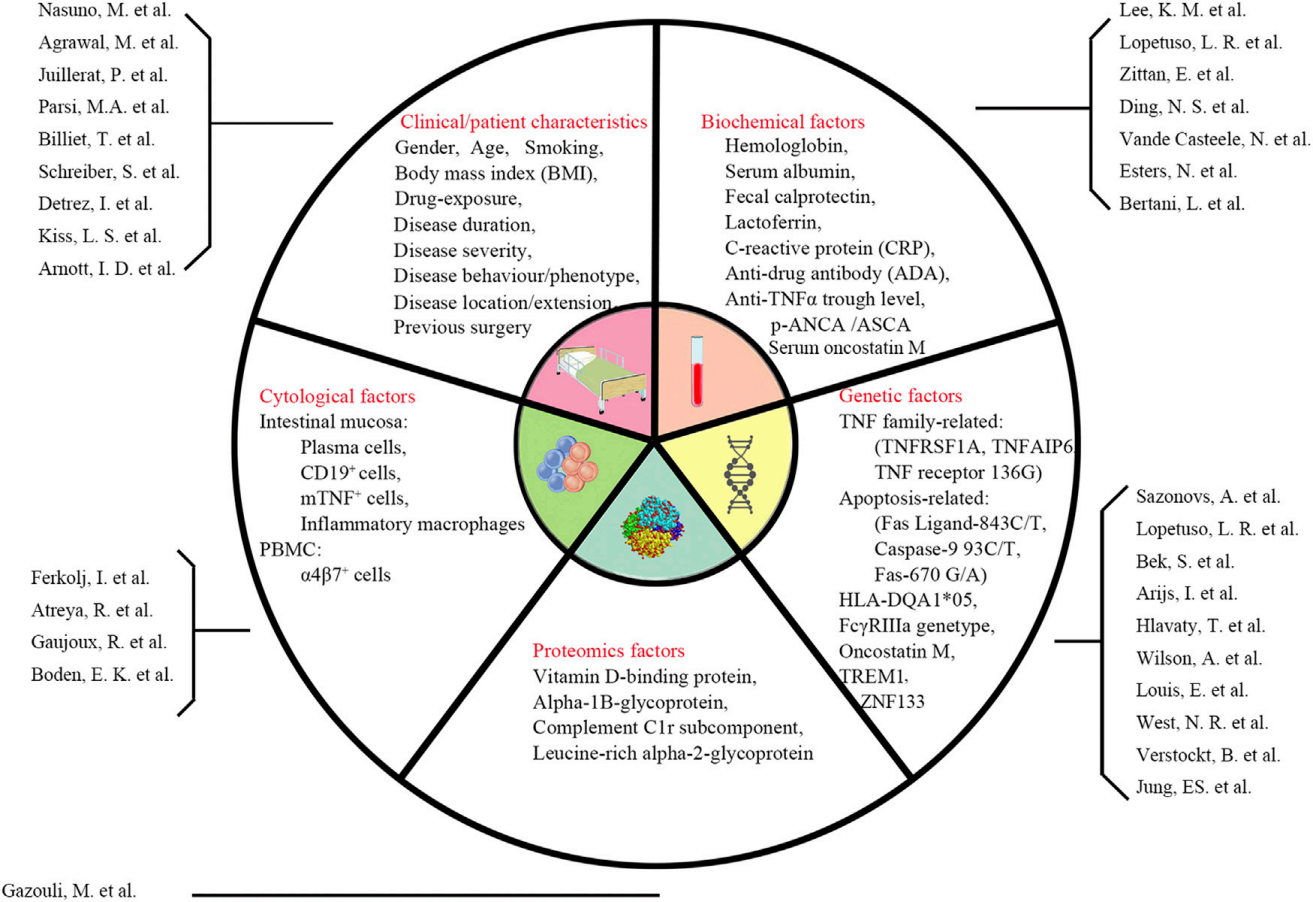
Summary of previous predictors of response to biologic agents (Esters et al., 2002; Parsi et al., 2002; Arnott et al., 2003; Louis et al., 2004; Ferkolj et al., 2005; Hlavaty et al., 2007; Arijs et al., 2010; Schreiber et al., 2010; Kiss et al., 2011; Gazouli et al., 2013; Lee et al., 2013; Atreya et al., 2014; Billiet et al., 2015; Juillerat et al., 2015; Vande Casteele et al., 2015; Bek et al., 2016; Detrez et al., 2016; Ding et al., 2016; Zittan et al., 2016; Lopetuso et al., 2017; Nasuno et al., 2017; West et al., 2017; Boden et al., 2018; Gaujoux et al., 2019; Jung et al., 2019; Verstockt et al., 2019; Wilson et al., 2020; Agrawal et al., 2022; Bertani et al., 2022)

Over the past few decades, the issue of predicting the response of patients with IBD to biologics has been of great concern. Clinical, biological, and genetic indicators have been measured, but to date, no predictor of high clinical value has been identified (Pariente et al., 2012; Tighe et al., 2017; Karmi et al., 2021) (Figure 2). Therefore, predictors or models with high specificity and sensitivity are needed to guide the selection of biologics for IBD treatment.

#### 4.1.2 Novel Predictive Models Encompassing Gut Microbes Mark a New Era in Biological Therapy

Considering the numerous changes in gut microbiota during biological therapy, we increasingly believe that responders and non-responders can be distinguished based on their differences in gut microbiota composition and metabolic levels. Extensive studies have been conducted to identify valuable biomarkers associated with gut microbes.

A clinical study on predicting week 30 remission in patients with CD treated with infliximab revealed that compared with the Crohn’s Disease Activity Index (CDAI; 58.7% predictive accuracy) and calprotectin (62.5% predictive accuracy), which are commonly used for disease evaluation in clinical practice, the use of certain gut microbes, mainly Clostridiales, increased the predictive accuracy to 86.5% alone and 93.8% combined with the data of CDAI and calprotectin (Zhou et al., 2018). Another study revealed that the enrichment of Lachnospiraceae and *Blautia* at week 6 of infliximab treatment correlated with clinical response, and the combined increase of these taxa showed 84.2% and 89.1% accuracy in predicting clinical and endoscopic responses at weeks 14 and 30, respectively (Zhuang et al., 2020). Furthermore, even when used as predictors alone, gut microbiota also exhibited a higher value (AUC, 0.849) compared to clinical data (AUC, 0.624) (Lee et al., 2021).

Vedonet, a neural network algorithm that combines the microbiome and clinical data, was found to be effective in predicting the efficacy of vedolizumab, with a true positive rate of 87.2% and a false negative rate of <25%, and was validated in another anti-TNFα cohort, also demonstrating a high predictive value (Ananthakrishnan et al., 2017). In 2018, Doherty et al. achieved high prediction levels of 83.1% specificity and 77.4% sensitivity in ustekinumab therapy for CD based on a random forest model containing gut microbes and clinical indicators, which was significantly higher than prediction levels using only clinical data (Doherty et al., 2018). Recently, predictive models designed on the basis of SCFA-producing bacteria *Enterobacter*, *Streptococcus* and *Granulicatella*, have also been proven to reach a high level of prediction (area under the curve >0.8) encouragingly (Ventin-Holmberg et al., 2021). Furthermore, even when used as predictors alone, gut microbiota also exhibited a higher value (AUC, 0.849) compared to clinical data (AUC, 0.624) (Lee et al., 2021).

As shown in Table 1, by comparing different predictors or models, it was found that the predictive models incorporating gut microbes improved the accuracy of prediction markedly, indicating the promising application of gut microbes in predicting the efficacy of biologics.

**TABLE 1:** Comparison of predictive effects of different prediction models with or without gut microbiota.

	Study author	Biologics	Disease	Specimen	Design	Research technique	Population (cohort size)	Predictive markers	Prediction accuracy/AUC	Prediction time
Prediction model with gut microbiota	Arijs et al. (2009)	IFX	UC	Mucosal biopsy	cross-sectional	microarray and qPCR	cohort A: adult (24)	top five genes from cohort A	accuracy:A to A (83%) A to B (59.1%)	week 4–6
							cohort B: adult (22)	top five genes from cohort B	accuracy:B to B (90.9%) B to A (70.8%)	Week 8
	Billiet et al. (2015)	IFX	CD	clinical data	retrospective	Matrix model	adult CD (201)	age at first IFX, BMI, and previous surgery	0.78 < AUC <0.80	week 14
	Gonczi et al. (2017)	IFX	IBD	serum	prospective	ELISA	adult CD (184)	Infliximab trough level	AUCTLweek2 = 0.72	week 14
							adult UC (107)	Infliximab trough level	AUC TLweek2 = 0.81	week 14
	Bar-Yoseph et al. (2018)	IFX	IBD	serum	retrospective	ELISA	adult (140)	Infliximab levels <6.8 μg/ml ATI > 4.3 μg/mL-eq	AUC = 0.68	week 14
									AUC = 0.78	
	Dulai et al. (2018)	VDZ	CD	GEMINI 2 and VICTORY Dataset	cross-sectional	Model derivation	discovery cohort: GEMINI 2 (814) Validation cohort: VICTORY (336)	Individual multi-variable logistic regression prediction models	AUC = 0.67	week 26
	Zhou et al. (2018)	IFX	CD	serum, clinical data	prospective	ELISA	discovery cohort: adult (16) Validation cohort: RISK(668), PRISM(155)	CDAI	accuracy: CD (58.7%)	week 30
								Fecal calprotectin	accuracy: CD (62.5%)	
	Engström et al. (2019)	IFX	IBD	feces, serum	cross-sectional	ELISA and near-infrared particle immunoassay	adult (CD: 76 UC: 47)	Fecal calprotectin >221 μg/g	AUC = 0.71	week 12
								CRP > 2.1 mg/L	AUC = 0.58	
	Shi et al. (2021)	IFX/ADA	IBD	Mucosal biopsy	cross-sectional	RNA-seq and microarray	GEO and SRA databases	GIMATS module	AUC = 0.720–0.853	week 4–6
		VDZ							AUC = 0.661–0.728	
	Lee et al., 2021	IFX/UST/VDZ	IBD	feces, serum	prospective	Random forest classifiers	adult (CD: 108 UC: 77)	clinical features	AUC = 0.624	week 14
Prediction model with gut microbiota	Ananthakrishnan et al. (2017)	IFX,VDA	IBD	feces	prospective	16srRNA	adult (CD: 42 UC: 43)	Gut microbiota	AUC 0.872	week 14
	Zhou et al. (2018)	IFX	IBD	feces	prospective	16srRNA	Discovery cohart :adult (16) Validati Cohort: RISK (668) PRISM(155)	Gut microbiota	Accuracy.CD (87.5%) UC (79.1%)	week 30
	Zhou et al. (2018)	IFX	IBD	feces, serum,Clinical, data	prospective	16srRNA ELISA	Discovery cohart :adult (16) Validati Cohort: RISK PRISM(155)	Gut microbiota+FC+CDAI	Accuracy.CD (93.8%)	week 30
	Doherty (2018)	UST	CD	feces, serum,Clinical, data	prospective	16srRNA	Adult(306)	Gut microbiota	AUC = 0.844	week 6
	Zhuang et al, (2020)	IFX	CD	feces	prospective	16srRNA	Adult(49)	Gut microbiota	Clinical response (83.4%) Clinical response (83.4%) endoscopic response(89.1%)	week 30
	Ventin and Holmberg et al, (2021)	IFX	IBD	feces	prospective	16srRNA	adult (CD: 25 UC: 47)	Gut microbiota	CD AUC = 0.933 UC AUC=0.818	week 12
	Lee et al. (2021)	IFX/UST/VDZ	IBD	feces, serum	prospective	Metagenomic Sequencing	adult (CD: 108 UC: 77)	Gut microbiota+Clinical Features	AUC = 0.849	week 14

### 4.2 New Therapeutic Strategy: The Combination of Faecal Microbiota Transplantation (FMT) and Biologics

Numerous studies have shown that biological therapy, which is currently the main treatment strategy for IBD, does not fully restore all intestinal microbiota, including SCFA-producing bacteria and intestinal metabolism (Wang et al., 2018; Kowalska-Duplaga et al., 2020). This may be one of the reasons for the poor response to biologics. Moreover, the aggravation of side effects caused by repeated high doses due to poor responses have attracted increasing attentions. Therefore, we sought to identify adjuvant therapies that could increase the effectiveness of biologics and reduce side effects.

FMT, an emerging therapeutic modality, has been successfully applied in treating recurrent *Clostridium difficile* infections with high cure rates (80–90%). Furthermore, many new indications for FMT have emerged, including IBD, metabolic diseases, graft-versus-host diseases, and neurological diseases (Allegretti et al., 2019). The therapeutic value of FMT for IBD, especially UC, has been previously reported. Whether treated with single, double, or multiple FMT treatments, patients with active UC showed a good response to FMT, achieving steroid-free clinical and endoscopic remission at 7–8 weeks (Moayyedi et al., 2015; Costello et al., 2019; Fang et al., 2021). A network meta-analysis of 16 randomised controlled trials revealed that infliximab, vedolizumab, and FMT demonstrated good efficacy in the treatment of UC. There was no statistically significant difference in efficacy among the three treatment regimens, further suggesting the possibility of FMT as a promising and efficient alternative to biologic agents (Zhou et al., 2021). High safety is another advantage of FMT, with a significantly lower rate of clinical recurrence and serious adverse effects in patients treated with FMT compared to infliximab (Li et al., 2021b). Consequently, as a promising candidate, FMT has attracted a great deal of interest. We propose a combination of FMT and biologics as a promising therapy that may play a synergistic role in the treatment of IBD to improve the efficacy and reduce the side effects caused by repeated dosing of biologic agents.

#### 4.2.1 Therapeutic Mechanisms of the Combination of FMT and Biologics in IBD

After analysing the identified therapeutic mechanisms of FMT and biologics in IBD, we conclude that the combination of FMT and biologics can synergistically improve the efficacy of biologic agents by resetting the intestinal microbiota and metabolism, maintaining the intestinal mucosal barrier, and modulating intestinal immune responses.

##### 4.2.1.1 FMT Restores the Composition and Metabolism of Intestinal Microbiota, Contributing to a Good Response to Biologics

Based on both colon and faecal samples, numerous studies have demonstrated that FMT increases the microbial diversity and improves the intestinal microbiota structures (Paramsothy et al., 2019), which is closely associated with a good response to biologic agents. At the family and genus levels, therapeutic FMT increased the abundance of SCFA-producers, such as *Coprococcus, Bifidobacterium*, Ruminococcaceae and Lachnospiraceae, all of these taxa contribute to a better response to biologics. Meanwhile, bacteria such as *Shigella*, *Escherichia coli*, and *Bacteroides acidifaciens*, which are significantly upregulated in IBD and associated with poor response to biologics, were also reduced after therapeutic FMT (Kellermayer et al., 2015; Tian et al., 2016; Burrello et al., 2019).

In terms of functional metabolism, pathways associated with bacterial over proliferation and inflammatory response (e.g., phenylalanine metabolism, bisphenol degradation, fatty acid biosynthesis) are significantly reduced after FMT treatment, while the levels of tryptophan metabolism and amino acid metabolism associated with bacterial fermentation are significantly increased. Furthermore, FMT increases the levels of secondary bile acids and short-chain fatty acid synthesis, which have been demonstrated to be markedly downregulated in the intestine of patients with IBD. Notably, levels of both amino acid metabolism and short-chain fatty acid synthesis mentioned above are positively correlated with the effectiveness of biologic agents in IBD (Burrello et al., 2019; Paramsothy et al., 2019).

##### 4.2.1.2 FMT Contributes to the Maintenance of Intestinal Barrier Function and Acts Synergistically With Biologics

An efficient epithelial barrier consists of physical, cellular, and chemical components, and its damage leads to increased epithelial permeability and dysbiosis (Rescigno, 2011). Among the components of the intestinal epithelial barrier, the tight junctions between intestinal epithelial cells, antimicrobial peptides secreted by Paneth cells, and loose mucus layer composed of mucin glycoproteins which are secreted by absorptive enterocytes or goblet cells, are essential for the intestinal mucosal barrier (Daneman and Rescigno, 2009). In both chronic and acute enteritis mouse models, the expression of tight junction proteins ZO-1, mucin genes, and antimicrobial peptide genes was restored to normal levels after FMT treatment, indicating an improvement in intestinal barrier function (Burrello et al., 2018; Burrello et al., 2019).

Correspondingly, biologic agents also have a positive effect on maintaining intestinal barrier function and promoting mucosal repair. Anti-TNFα agents maintain the effectiveness of the intestinal mucosal barrier and promote mucosal healing by reducing epithelial cell apoptosis, protecting tight junctions between epithelial cells, inhibiting intestinal vascular inflammation, and regulating myofibrillar function (Di Sabatino et al., 2007; Fischer et al., 2013). In addition, anti-TNFα agents induce the formation of regulatory macrophages, also known as M2-type macrophages or wound-healing macrophages, which express the cell surface marker CD206. This immunosuppressive cell population contributes to the repair of the intestinal mucosa (Vos et al., 2011; Vos et al., 2012; Schleier et al., 2020).

Therefore, in view of the common targets in therapeutic mechanisms, we suggest that the combination of FMT and biologics could play a synergistic role in mucosal repair and improve therapeutic efficacy.

##### 4.2.1.3 FMT and Biologic Agents Jointly Promote the Transformation of the Intestinal Mucosa From Inflammatory Mode to Anti-Inflammatory Mode

As a major component of the intestinal adaptive immune system, CD4^+^ T cells play a key role in orchestrating IBD-related inflammatory processes. Among CD4^+^ T cells, the balance between anti-inflammatory Treg cells and pro-inflammatory Th17/Th1 cells is closely associated with the progression and prognosis of IBD (Britton et al., 2019; Lee et al., 2020). Intestinal Treg cells maintain immune tolerance and suppress effector T cell-mediated immune injury (Barnes and Powrie, 2009; Honda and Littman, 2016). The inhibitory cytokine, IL-10 produced by Treg cells, antagonises the development of colitis (Lord, 2015; Cook et al., 2019; Wei et al., 2020). In contrast, pathogenic Th17 cells, also known as colitis-causing T cells, can release large amounts of the pro-inflammatory cytokines IL-17, IL-6, IL-22, TNF-α, IFN-γ, and GM-CSF (granulocyte-macrophage colony stimulating factor) after being activated by IL-23 and IL-1β, and can also help to promote the production of Th1 cells, further aggravating intestinal inflammation (Harbour et al., 2015; Honda and Littman, 2016; Jain et al., 2016).

**FIGURE 3:**
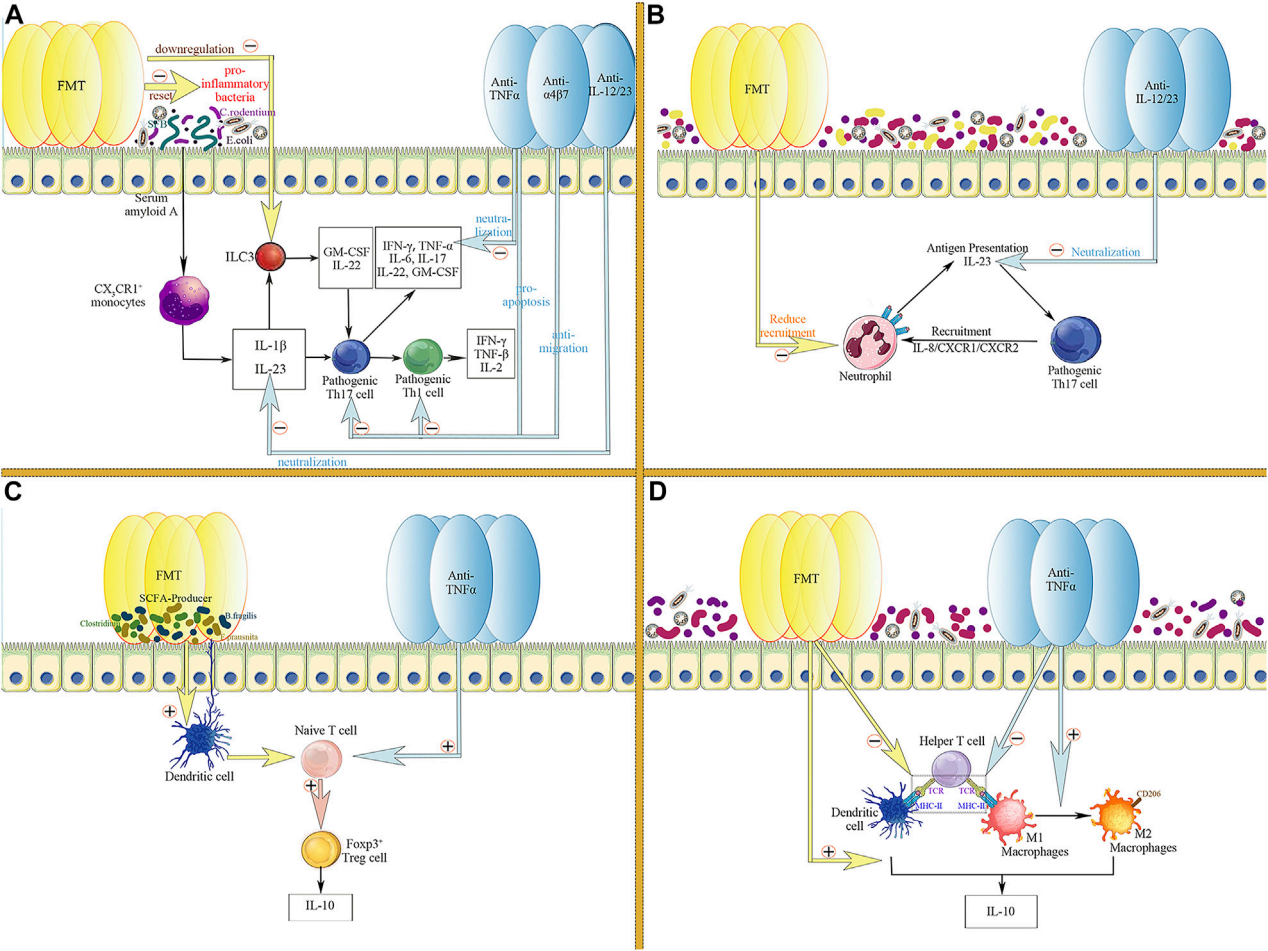
Common targets of FMT and biologic agents in the regulation of intestinal inflammatory responses. **(A)** FMT and biologics co-inhibit the activation and proliferation of pathogenic Th17 and Th1 cells; **(B)** FMT and biologics co-target the crosstalk between neutrophils and pathogenic Th17 cells; **(C)** FMT and biologics jointly promote the proliferation of Foxp3^+^ Treg cells and the formation of the anti-inflammatory cytokine IL-10; **(D)** FMT and biologics downregulate the function of helper T cells through inhibiting antigen presentation by APCs, including macrophages and dendritic cells, thereby suppressing the expansion of the inflammatory response; Meanwhile, FMT and biologics jointly promote the production of the anti-inflammatory cytokine IL-10 by APC.

Pro-inflammatory microbes, such as *Segmented filamentous bacteria*, *Citrobacter rodentium*, and *Escherichia coli*, are known to promote the production of IL-1β and IL-23 by CX3CR1^+^ monocytes through upregulation of serum amyloid in the intestinal epithelium (Bauché et al., 2018; Lee et al., 2020). IL-1β and IL-23 synergistically lead to further activation of pathogenic Th17 cells (Coccia et al., 2012; Atarashi et al., 2015; Lee et al., 2020). Pro-inflammatory microbes can also activate ILC3 with the aid of IL-1β and IL-23 to produce GM-CSF and IL-22, further activating pathogenic Th17 cells (Honda and Littman, 2016). In contrast, therapeutic FMT exogenously resets disturbed intestinal microecology in the inflammatory intestinal environment of patients with IBD. Thus, the abundance of pro-inflammatory bacteria and the frequency of ILC3 is decreased, thereby the activation of pathogenic Th17 cells is inhibited, and the production of Th1 cells and related inflammatory cytokines is reduced (Burrello et al., 2018). In addition, FMT also downregulates long-chain fatty acids (LCFA) in the inflamed intestine, thereby reducing the proliferation and activation of pathogenic Th17 cells stimulated by LCFA (Honda and Littman, 2016). Interestingly, various biologic agents also target the proliferation and activation of Th17/Th1 cells and the production of inflammatory cytokines. Anti-TNFα agents reduce the level of the pro-inflammatory cytokine TNFα in the inflammatory microenvironment by neutralising and inducing rapid apoptosis of Th1 and Th17 cells through direct, indirect, and Fc-dependent pathways (Levin et al., 2016). Consistently, vedolizumab inhibit the recruitment and proliferation of CD4^+^ T cells (especially Th1 and Th17 cells) by reducing the migration of T cells into the intestinal lamina propria (Feagan et al., 2013). Furthermore, ustekinumab directly neutralised the pro-inflammatory cytokine IL-23, reducing the proliferation and activation of pathogenic Th17 cells (Ahern et al., 2010) (Figure 3A).

In addition, FMT and biologics can jointly target the crosstalk between Th17 cells and neutrophils infiltrating the intestinal lamina propria to alleviate intestinal inflammation (Pelletier et al., 2010). In the colonic lamina propria of patients with IBD, Th17 cells highly express IL-8 and mediate the recruitment of tissue-infiltrating neutrophils, which have been proven to be the main producers of IL-23 in the intestine in an IL-8/CXCR1/CXCR2-dependent manner (Kvedaraite et al., 2016; Kvedaraite, 2021). These tissue-infiltrating neutrophils, in turn, present antigens and activate pathogenic Th17 cells with the aid of IL-23, thereby building a pro-inflammatory microenvironment and maintaining a sustained inflammatory response in the intestine (Kvedaraite, 2021). FMT decreased the number of neutrophils in the mucosal lamina propria, thereby reducing IL-23 production and antigen presentation to Th17 cells (Burrello et al., 2018). Similarly, the direct neutralising effect of ustekinumab, a monoclonal antibody targeting the shared p40 subunit of IL-12 and IL-23, also contributes to the downregulation of IL-23 and reduces the activation of pathogenic Th17 cells (Teng et al., 2015; Feagan et al., 2016) (Figure 3B).

The modulation of Treg cells is another common target of FMT and biologics. SCFA producers (e.g., *Bacteroides fragilis*, *Odoribacter splanchnicus*, *Faecalibacterium prausnitaii*, and *indigenous Clostridium*) can induce Treg cell production in the colonic lamina propria (Atarashi et al., 2011; Wei et al., 2020). Therefore, therapeutic FMT can alleviate colonic inflammation by increasing the abundance of SCFA-producing bacteria, thus promoting IL-10 production by Treg (Tian et al., 2016). Likewise, TNFα-targeting biologics also enhanced the number and function of Foxp3^+^ regulatory T cells in the peripheral blood and lamina propria of the intestinal mucosa during IBD treatment (Ricciardelli et al., 2008; Boschetti et al., 2011; Li et al., 2015), having a synergistic effect with FMT (Figure 3C).

Furthermore, professional antigen-presenting cells (APCs) are essential for the initiation and progression of intestinal inflammation. APCs promote inflammation by releasing cytokines upon antigen recognition and trigger a more intense immune response by presenting antigens and activating Th cells (Kaser et al., 2010; Neurath, 2014). After therapeutic FMT, the number of professional APCs (dendritic cells [DCs] and macrophages) and the expression level of MHC-II on the APCs were significantly diminished. FMT reversed the cytokine profile of antigen-presenting cells. Intestinal DCs and monocytes tend to produce more IL-10, suggesting that FMT inhibits colitis by downregulating antigen presentation and promoting the production of IL-10 (Burrello et al., 2018). Interestingly, two other non-negligible targets of biologic agents are the modulation of APCs and the induction of phenotypic switch of M1 to M2 macrophages, thereby downregulating antigen presentation and promoting IL-10 production (Baldwin et al., 2010; Vos et al., 2011; Billmeier et al., 2016) (Figure 3D).

Accordingly, through the common targets of pathogenic Th17/Th1 cells, neutrophils, regulatory T cells and professional antigen-presenting cells, FMT and biologics can jointly improve the inflammatory immune environment in the intestine. Furthermore, the synergy between FMT and biologics in the treatment of IBD provides a mechanistic basis for the feasibility of combination therapy.

#### 4.2.2 Advantages and Challenges of FMT Combined With Biologic Agent Therapy

The vicious circle between gut dysbiosis and the overactive intestinal mucosal immunity induces uncontrolled intestinal inflammation. Notably, gut dysbiosis occurs prior to the clinical and pathological manifestations of IBD, as has been demonstrated in first-degree relatives of patients with familial IBD (Jacobs et al., 2016). In the new therapeutic strategy, FMT directly targets intestinal dysbiosis, which acts in the upstream stages of IBD pathogenesis, providing a healthier intestinal ecosystem for biological therapy, thereby helping to maintain the efficacy of biologic agents and reduce the incidence of secondary non-response. Meanwhile, the application of biologic agents improves the inflammatory immune environment in the gut, providing the necessary biochemical basis for the colonisation of healthy intestinal microbiota, which is more conducive to improving the efficacy of FMT (Chu et al., 2021; Barron and Young, 2022). Additionally, given the multiple common targets of FMT and biologics in the treatment of IBD, the synergy between FMT and biologics jointly improves the therapeutic effect in patients with IBD and reduce the adverse effects associated with increased doses of biologics due to the poor response. Of note, secondary bacterial infections of the intestine, as one of the adverse effects of biologic agents, can also be prevented by the transfer of healthy gut microbes, due to the colonisation resistance provided by the transferred gut microbiota against pathogens (Shivaji et al., 2019; Alagna et al., 2020; Zhang et al., 2022). Therefore, compared with monotherapy of biologic agents, the new combined therapeutic strategy that includes FMT will largely improve the efficacy of treatment and benefit patients to a large extent.

Nevertheless, this new combined therapeutic strategy also faces challenges. As an emerging treatment modality, it remains difficult to programmatically specify the protocol of FMT, including the acquisition and preparation of the transferred material, etc. The resulting potential instability in the efficacy of FMT treatment is a source of concern (Danne et al., 2021). Furthermore, even though large clinical studies have demonstrated the broad safety of FMT, the possibility remains that some potentially harmful microorganisms and functions may be transferred by this treatment modality (Kassam et al., 2013). In particular, considering the upsurge of antibiotic resistance, FMT has the potential to mediate the transfer of antibiotic resistance genes and associated virulence factors to recipients, leading to some unintended consequences (Chu et al., 2021; Marcella et al., 2021).

Consequently, as an extremely promising combination treatment option, the therapeutic strategy of FMT combined with biologics offers great advantages in the treatment of IBD; however, there are many challenges (Sorbara and Pamer, 2022). Larger clinical trials are required to clarify the clinical indications, treatment regimens, and disease management to avoid potential risks.

## 5 Conclusion and Perspectives

The intestinal microbiota is closely correlated with the development of IBD and shows significant individual variations in responses to biologic agents. As mentioned above, recent studies have validated predictive models based on gut microbiota, clinical data, and serologic markers that have a higher accuracy on biologics’ therapeutic efficacy than traditional biomarkers. Nevertheless, one concern is that the number of such studies is relatively small. Another issue is the heterogeneity of the study design. For example, differences in criteria for defining biological response, length of follow-up, sample size, race, disease subtype, and type of biologic agents can all affect the generalisability of predictive models. Although there are no definitive models of the dominant gut microbiota that predicts the efficacy of biologics, according to Figure 1, we conclude that the abundance of Clostridia and its downstream taxa is positively correlated with a good biological response. In contrast, the abundance of Proteobacteria and the vast majority of its downstream taxa are negatively correlated with a good biological response.

Meanwhile, growing evidence shows that a more refined classification of gut microbes contributes to higher predictive accuracy. Classification at the class or family level does not perform as well as classification at the species or genus level. Therefore, future predictive models should incorporate information on taxa at species and genus levels. In the future, a comprehensive model with a higher predictive efficacy will require the integration of multi-omics information (including microbiomics, metatranscriptomics, metabolomics, and proteomics), clinical data, and serological markers from patients to optimise treatment strategies for IBD and deliver personalised therapeutic regimens.

By reviewing the common targets of FMT and biologics in the mechanisms of IBD treatment, we highlighted that the combination of FMT with biologics was a promising therapeutic strategy to increase the effectiveness of IBD treatment and reduce the rate of non-response and adverse effects of biologics. However, this particular therapy poses some issues. First, as an emerging therapeutic approach, since there are no treatment guidelines for FMT, different treatment strategies, such as the definition of indications, choice of co-administered biologics, selection of donors, use of antibiotics, mode and frequency of administration, and preparation and storage of faecal material, can result in different treatment outcomes. Additionally, FMT has raised challenges for regulators. For example, whether faecal suspensions, as a specific therapeutic material, ought to be regulated as a medicinal product or only as a medical practice, how to manage licences related to the production of the material, and how to regulate the potential transmission of infectious diseases or other unknown risks related to changes in the microbiota. Furthermore, more in-depth mechanistic studies are needed to explore the relationship between gut microbiota and biological therapy for IBD, which may provide more meaningful therapeutic insights.

In conclusion, as an emerging research area in IBD, gut microbiota is closely associated with traditional biological therapy. In future personalised treatment models of IBD, the combination of gut microbiota and biologics will show broader application prospects.
